# Advances in SXFA-Coated SAW Chemical Sensors for Organophosphorous Compound Detection

**DOI:** 10.3390/s110201526

**Published:** 2011-01-27

**Authors:** Wen Wang, Shitang He, Shunzhou Li, Minghua Liu, Yong Pan

**Affiliations:** 1 Institute of Acoustics, Chinese Academy of Sciences, Beijing, 100190, China; E-Mails: heshitang@mail.ioa.ac.cn (S.H.); lishunzhou@yahoo.com (S.L.); liuminghua@mail.ioa.ac.cn (M.L.); 2 Research Institute of Chemical Defense, Beijing, 102205, China

**Keywords:** SAW chemical sensor, response mechanism, oscillator, threshold detection limit

## Abstract

A polymer-coated surface acoustic wave (SAW)-based chemical sensor for organophosphorous compound sensing at extremely low concentrations was developed, in which a dual-delay-line oscillator coated with fluoroalcoholpolysiloxane (SXFA) acted as the sensor element. Response mechanism analysis was performed on the SXFA-coated chemical sensor, resulting in the optimal design parameters. The shear modulus of the SXFA, which is the key parameter for theoretical simulation, was extracted experimentally. New designs were done on the SAW devices to decrease the insertion loss. Referring to the new phase modulation approach, superior short-term frequency stability (±2 Hz in seconds) was achieved from the SAW oscillator using the fabricated 300 MHz delay line as the feedback element. In the sensor experiment on dimethylmethylphosphonate (DMMP) detection, the fabricated SXFA-coated chemical sensor exhibited an excellent threshold detection limit up to 0.004 mg/m^3^ (0.7 ppb) and good sensitivity (∼485 Hz/mg/m^3^ for a DMMP concentration of 2∼14 mg/m^3^).

## 1. Introduction

**Figure 1.**
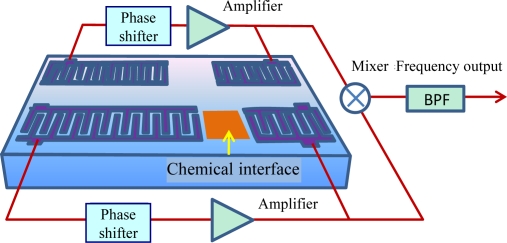
The schematic and principle of the polymer-coated SAW chemical sensor.

Due to the threat of terrorism and environmental pollution there is great demand for chemical sensors with high sensitivity and good stability towards organophosphorous compounds for real-time monitoring. Among currently available chemical sensors, surface acoustic wave (SAW) devices are favored and are very promising for chemical sensing applications due to their small size, low cost, high sensitivity, and reliability. Figure 1 shows the schematic and working principle of a typical SAW chemical sensor, composed of a dual-delay-line oscillator and a chemical interface coated onto the acoustic path of a SAW device. SAW chemical sensors present variations of the SAW phase velocity and attenuation as the vapor adsorbed on the chemical interface. The chemical interface is a chemical compound over the SAW propagating path, that selectively and reversibly interacts with the specific analyte vapor. The shift in phase velocity, and attenuation is measured by recording the frequency and insertion loss of the SAW device, respectively. Various effects, including mass loading, viscoelastic effect loading, and acousto-electric coupling [1–4], contribute to the SAW vapor response.

As well known, polymer materials are the primary chemical interface for vapor detection. Polymers have a higher sensitivity, lower detectable limits, and better ability to operate at room temperature than metal-oxide films [5]. Thus, the so-called viscoelastic effect loading contributes mainly to SAW due to the viscoelastic nature of polymers, in addition to the mass loading from the polymer deposition [6,7]. Usually, a bulk modulus *K* and a shear modulus *G* can be used to specify the mechanical properties of a linear and isotropic polymer. They are both complex, and their real parts (*G′* and *K′*) represent the storage moduli, where the imaginary parts (*G″* and *K″*) represent the loss moduli. A polymer with large shear modulus (G′ > 10 GPa) and G″ << G′ is a glassy (elastic) one. The rubbery (viscoelastic) regime is characterized by G′ ≤ 100 MPa, with G″ comparable to or less than G′. The glassy-rubbery polymer means that the polymer with a G′ which is 100 MPa < G′ < 10 GPa. Martin *et al.* first reported the response of polymer-coated SAW devices to temperature changes and polymer vapor absorption based on the perturbational approach [8]. Two different theory models were developed to predict velocity and attenuation induced by different polymer types. Grate *et al.* described the solubility interactions and the design of chemically selective sorbent coatings for chemical sensors and arrays in detail [9]. Kondoh *et al.* performed an optimization of the properties and thickness of polymers and the operating frequency theoretically [10]. Grate described the original motivation and principle behind the use of hydrogen-bond acidic polymers on chemical sensors and reviewed the types of polymer developed [11]. Yu-tang Shen *et al.* investigated the design rules for polymer-based ST-cut SAW sensors used in detecting organophosphorous compounds [12]. Calculations indicate that the glassy-rubbery film is most suitable in sensing application because it provides a larger and approximately linear sensing signal. However, the previous response mechanism analysis were aimed at chemical sensors based on traditional polymer deposition techniques like spin-casting, air-brushing, or dip-coating. Recently, some advanced techniques such as self-assembly (SEM) and molecularly imprinted (MI) technology are reported for polymer coating [13,14], in which, an active surface gold film between the sensitive film and substrate was used, the same requirement was also applicable to some simple polymer deposition techniques like solvent evaporation. Wang *et al.* presented some meaningful advances in sensor response mechanism analysis considering the effect of the metal film under such case, optimal design parameters like polymer thickness, and operation frequency were extracted theoretically [15].

Additionally, the use of SXFA (fluoroalcoholpolysiloxane) was frequently reported for chemical interfaces for organophosphorous compound detection due to its lower crystallinity and glassy transition temperature, and higher permeability [16,17]. However, despite some reported success stories, there still exists the problem of the extraction of optimal sensor parameters theoretically due to a lack of precise extraction of mechanical parameters like shear modulis. In this paper, the Martin model was used to deal with the response analysis of a SXFA-coated chemical sensor, its shear modulus was determined experimentally by the inversion method. Then, referring to the Wang model [15], the response mechanism of the SXFA-coated chemical sensor was depicted in detail, including the polymer thickness and frequency effect on vapor adsorption, allowing optimal design parameter extraction.

Also, it is well known that the sensor performance, especially the threshold detection limit, depend, mainly on the frequency stability of the oscillator. Thus, improvement of the frequency stability is another main topic of SAW chemical sensor research. Many groups have reported some success with SAW oscillators of different design for gas sensing. Schickfus *et al.* analyzed the effect of temperature changes, ageing of the transducer and the layer material on the frequency stability of the oscillator [18]. Hoyt *et al.* presented a way to improve the frequency stability of the oscillator, in which both of the dual delay line oscillators were modified with chemically sensitive interface materials to compensate for the noise and drift in SAW oscillator frequency and frequency associated with temperature effects on wave velocity in coating films [19]. Schmit *et al*. reported a rapid design of SAW oscillator electronics for sensor application with high frequency stability [20]. Jasel *et al.* reported the detailed design of oscillation circuits to improve the frequency stability of the oscillator [21]. Also, in our previous work, a new SAW device for chemical sensing design was done, in which electrode width controlled single-phase unidirectional transducers (EWC/SPUDT) and combed transducers were used to structure the SAW delay line, resulting in lower insertion loss and single oscillation frequency mode [13,14]. The frequency stability was improved effectively, however, to maintain the stable oscillator status, further work should be done for the oscillator including the SAW devices and oscillation circuit. This is the second topic in this paper. Here, a delay line with frequency of 300 MHz and Al/Au metallization was fabricated; lower insertion loss less than 10 dB and single frequency oscillation were realized from the measured data. A new approach of phase modulation was also demonstrated, which make the oscillation occur at the lowest insertion loss point. Superior short-term freqency stability was obtained experimentally. Next, the fabricated oscillator with good stability results and superior threshold detection limits, which was confirmed in the gas sensing experiment, in which, the SXFA with optimal thickness was used as the sensor material for DMMP detection.

## 2. Theoretical Analysis on Response Mechanism Analysis

**Figure 2.**
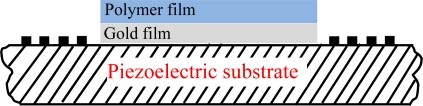
The schematic of the sensor structure and coordination system in this study.

To extract the optimal design parameters, the response mechanism analysis was performed on the SXFA-coated SAW chemical sensor using the theoretical model of by Wang *et al.* [15], including the polymer thickness effect and frequency effect on vapor adsorption. Considering the perturbation to SAW propagation along the sensor structure as shown in Figure 2 (including metal thin film and polymer on the top of the substrate) targeting the new polymer deposition technique described in Reference [15], the response formula of the polymer-coated chemical sensor could be given by Equation (1):

(1) $$\begin{array}{l} \Delta \beta /k_{0}=\Delta \alpha /k_{0}-j\Delta V/V^{\prime }=j\sum_{i}^{3}c_{i}^{\prime }\frac{\alpha_{i}M^{i}}{\omega }\cdot \text{tan}\left(\alpha_{i}\left(h_{0}\right)\right)\\ \alpha_{i}=\omega \sqrt{\frac{\rho_{0}-E^{i}/V^{\prime 2}}{M^{i}}}\\ E^{1}=\frac{4G(3K+G)}{3K+G},E^{2}=G,E^{3}=0,\\ M^{1}=M^{2}=G,M^{3}=K\\ j=\sqrt{-1} \end{array}$$

where Δ*β*/*k*_0_ expresses the fractional perturbation of the complex wave propagation factor *β* per wavenumber *k*_0_. Δ*α*/*k*_0_ is the change in attenuation α per *k*_0_, Δ*V* is the factional change in propagation velocity *V*′ after metal film coating. The *c*_i_′ (*I* = 1∼3) are SAW-film coupling parameters perturbed by the metal film. *ρ*_0_ is the polymer density. *G* and *K* are the shear modulus and bulk modulus, respectively. h0 is the polymer thickness. Using Equation (1), the changes of the velocity and attenuation resulting from the coated polymer can obviously also be obtained. Additionally, the thickness, *h*, and density, *ρ*, of the polymer are taken as function of the concentration of gas sorbed, *C*, as shown in Equation (2):

(2) $$\begin{array}{l} \rho (C)=\rho_{0}+\mathrm{CM}/(1+\mathrm{CU})\\ h(C)=h_{0}(1+\mathrm{CU}) \end{array}$$

where, *U* and *M* are specific volume and molecular weight of the sorption species, respectively. *C* and *U* in Equation (2), can be expressed as:

(3) $$\begin{array}{l} C={\kappa c}_{v}/M\\ U=M/\rho_{v} \end{array}$$

where *c_v_* is the vapor concentration, *ρ_v_* is the density of the vapor. The *κ* is the partition coefficient. Then, substituting Equation (3) into Equation (2), the polymer thickness and density depending on vapor adsorption can be described as:

(4) $$\begin{array}{l} h\left(c_{v}\right)=h_{0}\times \left(1+\frac{\kappa \times c_{v}}{\rho_{v}}\right)\\ \rho \left(c_{v}\right)=\frac{\rho_{0}+\kappa \times c_{v}}{1+\kappa \times c_{v}/\rho_{v}} \end{array}$$

Thus, the velocity shift and loss change due to gas sorption can be expressed as a function of the concentration of absorbed species by substituting Equation (4) into Equation (1).

### 2.1. Shear Modulus Determination of SXFA

Prior to the response simulation on gas adsorption, the shear modulus of the SXFA was determined experimentally. It is well known that the modulus of the polymer is very frequency dependent. However, in our study, the operation frequency of the chemical sensor is targeted to several hundred MHz. In such frequency range, the modulus is considered to independent on the frequency [8]. The network analyzer and ten fabricated SAW delay lines were prepared for the experiment. The SAW devices used in this study consisted of an ST-X quartz substrate with two photolithographically defined Cr/Au (30/200 nm) transducers separated by a path length (transducer center separation) of 6.4 mm (320 wavenumbers). Two transducers consist of 270 and 80 finger-pairs of interdigitated electrodes, with periodicity of 20 μm for a center frequency of 158 MHz. EWC/SPUDT and combed transducer were used to structure the transducers, which lead to low insertion loss and stable SAW oscillator for gas sensing [14].

Then, SXFA layers with different thicknesses were deposited onto the quartz surface of the fabricated SAW delay line by solvent-evaporating as described below. Here, the SXFA was synthesized by reaction of polyallylmethylsiloxane and hexafluoroacetone (HFA). Also, the thickness was estimated approximately by atomic force microscopy (AFM). Meanwhile, using the Equation (5) and the measured frequency shift and loss change arising from the acoustically thin SXFA coatings monitored by the network analyzer, the shear modulus of the SXFA can be deduced as shown in Table. 1. Here, the bulk modulus in soft, elastic solids is typically much larger than the shear modulus, thus, the perturbation to SAW by polymer can be expressed by the shear modulus *G*:

(5) $$\begin{array}{l} \Delta f/f_{0}≅-\omega h\left(\left(c_{1}+c_{2}+c_{3}\right)\times \rho -\left(c_{1}+4\times c_{3}\right)/v_{0}^{2}\times G\prime \right)\\ \mathrm{Loss}/54.6\times N_{A})≅\omega h/v_{0}^{2}\times \left(c_{1}+4\times c_{3}\right)\times G\prime \prime \end{array}$$

where, *c*_1_, *c*_2_ and *c*_3_ are SAW-film coupling parameters [8], *h* is the polymer thickness, *ρ* is the density of the polymer, the *ω* is angular frequency, *v*_0_ is unperturbed SAW velocity, *N_A_* is the acoustic path length (320 wavelengths in our design). The density of SXFA is 1.447 g/cm^3^. Next, using statistical method and inversion method, the shear modulus *G* of the SXFA can be evaluated as 9.3 × 10^9^ Pa + 1.2 × 10^9^ Pa. Hence, SXFA is the glassy-rubbery polymer materials according the classification as mentioned in the Introduction.

### 2.2. Effect of Polymer Thickness on Gas Adsorption

**Figure 3.**
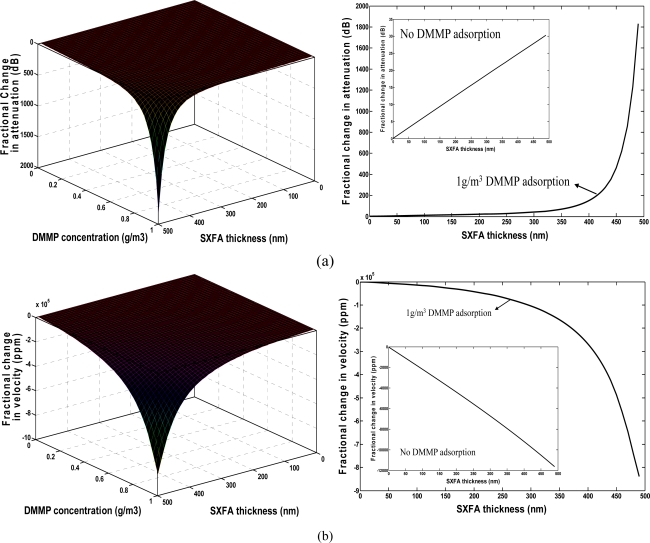
**(a)** Change in attenuation. **(b)** Velocity shift from SXFA on vapor adsorption.

Using the extracted shear modulus and by means of Equations (1–5), the effect of the polymer thickness on gas sensing was simulated as shown in Figure 3. The real part of the bulk modulus is assumed to be constant at 10 GPa. Moreover, the influence of the imaginary part of the bulk modulus can be neglected [12]. Also, the modulus is assumed to have no relevance to the operation frequency. A 300 nm gold thin film was assumed to coat onto the substrate surface prior to polymer deposition. The perturbed SAW velocity and SAW-film coupling parameters by gold film can be deduced using the related formula mentioned in reference [15]. The DMMP with density of 1.145 g/cm^3^ and molecular weight of 124 was assumed as the target species for the chemical sensor. The concentration of DMMP is set to 0∼1,000 mg/m^3^ in the simulation. The partition coefficient logκ of SXFA towards to DMMP is 6.4 [22,23].The operation frequency of the chemical sensor is assumed as 300 MHz. In Figure 3, the resonation phenomenon in velocity and loss change exhibits the viscoelastic nature of the SXFA. For a given vapor concentration range, the velocity shift is approximately a linear function of the SXFA thickness when the thickness is less than 300 nm. When thicker polymers are applied onto the SAW sensor, a resonance peak is observed as the film thickness increases. The plasticization and viscoelastic nature caused the polymer to behave like an acoustically thicker one. The strongest sensor response appears at very thick SXFA thickness, over 450 nm, however, a trade-off should be considered due to the fact this is to accompanied by much larger attenuation in Figure 3(a) during the DMMP adsorption. To keep stable oscillation in gas sensing, the induced attenuation from the polymer deposition should be reduced as much as possible, hence a thin SXFA film of less than 100 nm was advised in our work, which leads to linearly velocity change with large amplitude and lower attenuation (less than 10 dB) in the vapor adsorption.

### 2.3. Frequency Effect on Gas Adsorption

**Figure 4.**
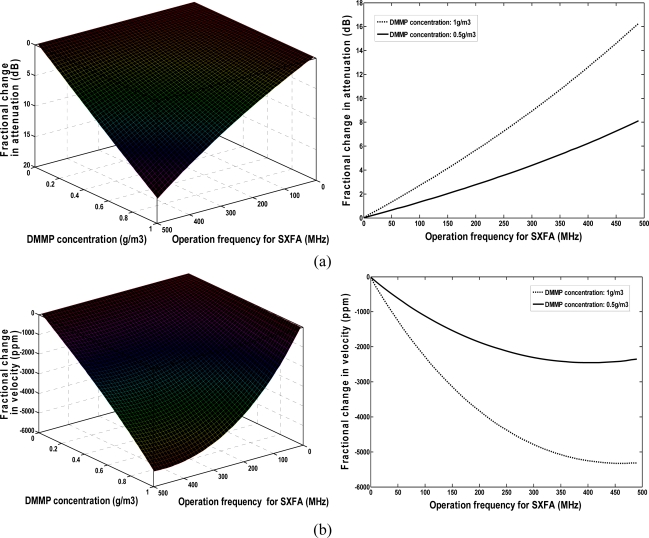
**(a)** Change in attenuation, and **(b)** velocity shift from frequency effect.

Figure 4 illustrates the relationships among the DMMP concentration (0∼1,000 mg/m^3^), operation frequency of polymer-coated sensor, velocity shift, and loss change under given SXFA thickness of 40 nm. The sensor response is approximately proportional to the given operation frequency in the assumed DMMP concentration range. The higher the operation frequency of the chemical sensor employed, the larger a sensor response will be achieved. However, similar to the polymer thickness effect analysis as above, higher attenuation was induced in the vapor adsorption when a higher frequency was applied. As shown in Figure 4, the largest sensor response towards to 1,000 mg/m^3^ DMMP was observed at max frequency of ∼500 MHz accompanying attenuation over 16 dB. Additionally, the advantageous sensor response-frequency dependence is hard to apply because of the decreased SAW device dimensions and thereby the area of the chemical interface with the operation frequency. Thus, the frequency of our SXFA-coated SAW sensor was set to 300 MHz, here, and good sensor response and a lower induced attenuation of less than 10 dB were observed.

## 3. SAW Oscillator Design

**Figure 5.**
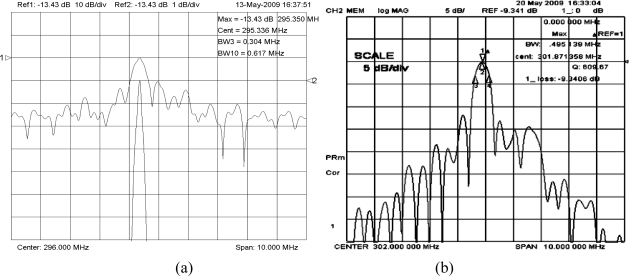
Measured S_12_ from SAW device with **(a)** Cr/Au, and **(b)** Al/Au electrodes.

**Figure 6.**
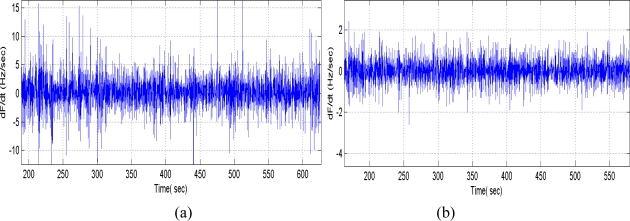
Frequency stability testing of oscillator using **(a)** Cr/Au, and **(b)** Al/Au electrode.

From the traditional Tiersten formula as Equation (6) [5], the sensor performance, especially the threshold detection limit, depend mainly on the frequency stability of the oscillator, which acts as the sensor elements:

(6) $$\Delta f/f_{0}=\left(c_{1}+c_{2}+c_{3}\right)\times f_{0}\times \Delta m/s$$

where, Δ*f* is the frequency response towards to gas adsorption with mass loading change of Δ*m*, *f*_0_ is the oscillation frequency, *c*_1_, *c*_2_ and *c*_3_ are the material constants of substrate, and s is the area of the chemical interface. Thus, improvement of the frequency stability is another important topic for the SAW chemical sensor. Recently, a SPUDT and combed transducers were reported to structure the SAW devices [10], which results in lower insertion loss, and single oscillation frequency. The reported SAW delay lines with operation frequency of 300 MHz consist of an ST-X quartz substrate with two photolithographically defined Cr/Au (30/200 nm) transducers separated by a path length (transducer center separation) of 2.5 mm. The two transducers consist of 520 and 160 wavelengths. A gold film with size of 2 × 2 mm^2^ was designed onto the quartz substrate between the transducers for polymer coating. Figure 5(a) shows the measured frequency response S_12_ of the SAW delay line with Cr/Au electrodes; an insertion loss of ∼13 dB was observed. Using the fabricated SAW devices as the feedback, a 300 MHz SAW oscillator was fabricated. Also, the oscillation occurred at the operation frequency of the SAW devices by adjusting the phase shifter. Here, the programmable frequency counter and computer are used to measure the frequency stability of the annealed SAW oscillator without a chemical interface on top of the substrate and no airflow. The testing conditions are temperature of 20 °C, and 68% RH. To demonstrate the short term frequency stability (seconds) distinctly, a partial differentiation was performed on the measured frequency shift to test time, it shows the frequency shift per second. Figure 6(a) indicates that the short term frequency stability was evaluated as frequency shift of ∼±15 Hz in seconds.

To make further improvement of the frequency stability, a new SAW delay line with Al/Au electrode structure was designed to decrease the insertion loss in this paper. As is well-known, the key parameter for the SPUDT design is the reflection coefficient of the electrode. Using the variational method and Campbell theory, the reflection coefficient of the Al/Au electrode was calculated depending on the fractional electrode thickness [24], also, the computation indicates the electromechanical constants was improved over the Cr/Au metallization, which was of benefit for the insertion loss reduction. On the basis of calculated results, a new 300 MHz delay line with SPUDT and combed transducer structure was designed and fabricated using the design parameters same as above devices in Figure 5(a) and Al/Au (30/200 nm) metallization. The S12 of the fabricated new device was tested by the network analyzer. As shown in Figure 5(b), a lower insertion loss of 9.3 dB was observed, obviously less than that of the delay line with Cr/Au metallization.

Using the fabricated new SAW devices as the feedback element and oscillation circuit consists of amplifier, phase shifter and mixer, a new dual-delay-line oscillator was realized. Also, the oscillation was modulated at the frequency point with lowest insertion loss by a strategically phase modulation, in which, a low pass filter acts as the phase shifter, the phase modulation was accomplished by adjusting the inductor or capacitor values. The measured short term frequency stability (frequency shift in seconds) was shown in Figure 6(b). Excellent short term frequency stability of ∼±2 Hz was observed, greatly better than that if the previous oscillator with similar structure [14,18].

## 4. Sensor Experiments

Based on the theoretical analysis of a polymer-coated SAW chemical sensor and new design on the SAW oscillation, a SXFA-coated SAW chemical sensor with operation frequency of 300 MHz in gas adsorption was realized using DMMP as the sensing species.

### 4.1. SXFA Deposition

**Figure 7.**
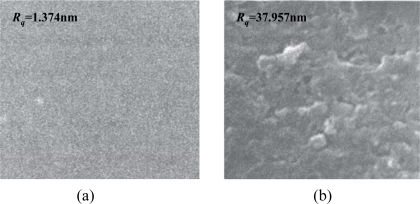
The SEM picture of **(a)** uncoated-surface, and **(b)** coated-surface of the delay line.

In our study, the solvent-evaporating method was used for the SXFA deposition, due to its simple operation and low cost. The effectiveness of the solvent evaporating method to produce a stable polymer coating depends on the selection of the solvent type. Here, toluene was used as the solvent. Before the deposition of the SXFA film, the gold surface between the transducers was cleaned of any contaminants by a routine cleaning procedure involving rinsing in piranha solution [V(H_2_SO_4_):V(H_2_O_2_) = 3:1], a DI water rinse and drying by N_2_. Then, a 0.1 μL solution of 0.8 g/L SXFA/toluene was deposited on the cleaned gold surface 10 consecutive times. From the theoretical analysis above, the SXFA thickness targeted was ∼40 nm. After SXFA coating, the coated surface was characterized by scanning electron microscope (SEM), as shown in Figure 7. The thickness of the SXFA coating was evaluated by the atomic force microscopy (AFM) which approximately represented the surface roughness. Before SXFA coating, the gold surface roughness is only 1.374 nm [Figure 7(a)], less more than the coated surface, in which, the surface roughness is evaluated as 37.957 nm [Figure 7(b)], similar to the predicted values. Also, the solvent-evaporating induces porous and amorphous SXFA deposition, which benefits stereospecific adsorption. Also, to verify the theoretical analysis of the polymer thickness effect, SXFA layers with different thickness (10∼100 nm) was coated onto the fabricated SAW devices.

### 4.2. Sensor Response Depending on SXFA Thickness

**Figure 8.**
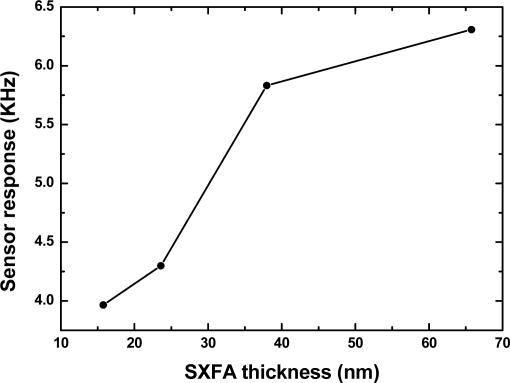
The measured effect of SXFA thickness on sensor response.

First, the fabricated SXFA-coated SAW sensors with different polymer thickness were applied to 200 mg/m^3^ of DMMP detection at 24 °C, 50% RH and gas flow speed of 1 L/min, as shown in Figure 8. From the picture, as the SXFA thickness increases, the sensor response towards to 200 mg/m^3^ DMMP increases as predicted. As mentioned in the theoretical analysis, thicker SXFA will result in a larger sensor response, however, higher attenuation will be induced accordingly, as was verified in our experiments. When a SXFA thickness over 70 nm was applied, the oscillator cannot work stably, and even stops working under larger vapor concentrations. This means the attenuation induced by the SXFA in gas adsorption was enough to hinder the oscillation. All the measured values verified the theory concernng the optimal extraction of polymer thickness mentioned in Section 2.

### 4.3. Temperature Effect on Vapor Adsorption

**Figure 9.**
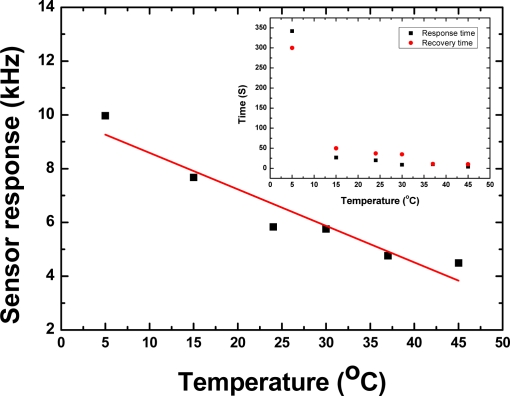
The temperature effect on sensor response and on response and recovery time.

It is well-known that the viscoelastic nature of the polymer depends on the testing temperature. When a higher testing temperature was applied, stronger viscoelastic properties and weaker hydrogen bonds were exhibited, resulting in deviation of sensitivity, response and recovery time and frequency response. Actually, the temperature properties of the polymer were determined by the glass transition temperature (*T_g_*). Using the differential scanning calorimetry (DSC) technique the *T_g_* of the SXFA was measured as ∼−44 °C, far less than room temperature. A DMMP adsorption experiment was performed for SXFA-coated sensor under different temperatures. Figure 9 shows the sensor response value of the SXFA sensor to 200 mg/m^3^ DMMP at different temperatures and RH of 50%.

Here, the gas concentration data was cross-validated by gas chromatography through field sampling analysis. From the picture, the sensor response towards to DMMP adsorption decreases increasing testing temperature. From 5 °C to 45 °C, the amplitude of sensor response decreases up to 55%. On the other hand, the response times are also influenced by the testing temperature, as shown in the inset of Figure 9. When a lower testing temperature was applied, a longer response time was observed. The response time to DMMP adsorption was over 342 s at a temperature of 5 °C, while a faster response time of less than 10 s was observed at a higher temperature of 45 °C. This means that closer to the *T_g_* of SXFA, a slower response time will be observed. These results are consistent with the expected slow diffusion of vapor in a glassy polymer. Moreover, the measured results illustrate the effect of the temperature on sensor response. Lower temperature induces slowing thermal motion of DMMP molecules, and hence slows the adsorbence process between the SXFA and DMMP molecules, resulting in longer response times. Also, the slow thermal motion and lower kinetic energy leads to a stronger interaction of DMMP molecules towards to SXFA coating. Thus, larger sensor response values and slow response times were observed at lower testing temperatures. The opposite phenomenon was observed for the sensor response at higher temperatures, it indicates lower sensitivity and fast response. Thus, a compromise must be considered for the testing temperature choice.

### 4.4. Repeatability Testing in Vapor Adsorption

**Figure 10.**
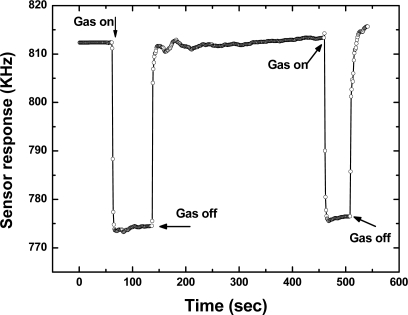
Repeatability testing of sensor responses to 1,000 mg/m^3^ DMMP.

Then, the repeatability of the fabricated SXFA-coated SAW sensor was investigated. Figure 10 showed a typical response profile obtained from two consecutive 100 seconds on-off exposures to 1,000 mg/m^3^ of DMMP at 24 °C, 68% RH and gas flow speed of 1 L/min. Here, the frequency response was recorded every 1 second so that one point on the graph corresponds to a 1-second interval. The sensor response showed a rapid rise upon exposure to DMMP and reaches the equilibrium (saturation) value in approximately 10 seconds. When the gas was removed by N_2_, the sensor response returned to its initial baseline value within 10 seconds. The transition of initial state-adsorption-stable equilibration state-desorption-recovery to initial state was clearly observed. From this promising result, we consider that this sensor has excellent repeatability in response to DMMP.

### 4.5. Sensitivity Evaluation

**Figure 11.**
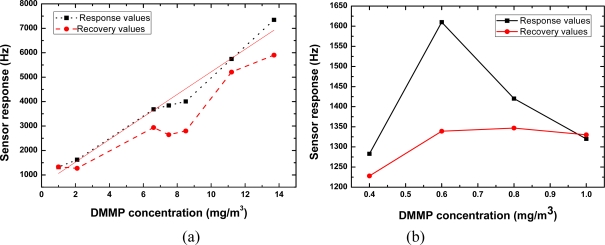
Sensor responses in terms of vapor concentration at **(a)** high, and **(b)** low DMMP concentrations

We exposed the SXFA-coated sensor to various DMMP concentrations to evaluate its sensitivity. Figure 11 shows the obtained frequency shifts at various DMMP concentrations under fixed 23% RH and 14 °C conditions. At high DMMP concentrations (>2.0 mg/m^3^), the sensor response increased linearly with increasing DMMP concentration, which satisfies the law of the adsorption isotherm in solids [Figure 11(a)]. Excellent reproducibility was also observed from the comparison between the response and recovery values. The sensitivity in the DMMP concentration range of 2∼14 mg/m^3^ was evaluated as ∼485 Hz/mg/m^3^. In contrast, at low DMMP concentrations (<1.0 mg/m^3^), the sensor response was complicated, as shown in Figure 11(b). In the initial part of the testing period, the sensor response increases with increasing concentration, however, the opposite phenomenon occurs upon further testing. A peak of the sensor response was observed at the DMMP concentration of 0.6 mg/m^3^, which is coincident with the adsorption isotherm in liquids. On the basis of the measured results, we suggest that at the tested temperature, viscous-liquid SXFA presents the adsorption properties of both solid and liquid forms. Also, from Figure 11(b), a larger sensor response (∼1,280 Hz) occurs at a DMMP concentration of 0.4 mg/m^3^. It means very lower threshold detection limit will be achieved using the present oscillator with superior frequency stability. In our sensor, based on the International Union of Pure and Applied Chemistry (IUPAC), it means the lower detection limit of 0.004 mg/m^3^ (0.7 ppb) is possible owing to the excellent short-term frequency stability of the oscillator (frequency shift of ±2 Hz in seconds), which provides a signal-to-noise ratio of more than 100:1. The measured data is better than the reported values from similar sensor structures [18]. The measured results indicate that the presented SXFA-coated chemical sensor was very promising for organophosphorous compound detection at extremely low concentration.

## 5. Conclusions

Advances in SAW chemical sensors relating to the response mechanism, design and fabrication of the SAW oscillator were described. The response mechanism of the SXFA-coated SAW chemical sensor extracts the optimal design parameters. New phase modulation methods and new design of the SAW device result a great improvement of the frequency stability of the SAW oscillator, with a measured result of up to ±2 Hz in seconds for the oscillator with an operation frequency of 300 MHz. Then, using the SXFA as the sensor material for DMMP detection, the fabricated SAW chemical sensor with 300 MHz was characterized in gas adsorption. Superior performance like high sensitivity (485 Hz/mg/m^3^), and a lower threshold detection limit of 0.004 mg/m^3^ (0.7 ppb) were obtained experimentally.

## Figures and Tables

**Table 1.** The extraction of the shear modulus of the SXFA.

**Device No.**	**Polymer thickness (nm)**	**Frequency before polymer coating (MHz)**	**Frequency change after polymer coating (MHz)**	**Loss change (dB)**	**G′ × 10^9^ (Pa)**	**G″ × 10^9^ (Pa)**
1	10.5	157.2875	−0.012	0.5374	9.0184	1.19
2	13.5	157.41875	−0.01325	0.6465	9.4333	1.1134
3	15	157.4	−0.015	0.7249	9.3857	1.1236
4	23	157.437	−0.0245	1.1507	9.218	1.1632
5	26	157.475	−0.027	1.4	9.2868	1.2525
6	30	157.437	−0.029	1.5	9.4714	1.1679
7	40	157.637	−0.04125	2.1329	9.3053	1.2397
8	45	157.20625	−0.045	2.329	9.3857	1.2033
9	65	157.2365	−0.0675	3.654	9.2868	1.307
10	87	157.497	−0.0875	4.545	9.3709	1.2146
